# Factors influencing health workers’ compliance with outpatient malaria ‘test and treat’ guidelines during the plateauing performance phase in Kenya, 2014–2016

**DOI:** 10.1186/s12936-022-04093-x

**Published:** 2022-03-03

**Authors:** Beatrice Amboko, Kasia Stepniewska, Beatrice Machini, Philip Bejon, Robert W. Snow, Dejan Zurovac

**Affiliations:** 1 KEMRI-Wellcome Trust Research Programme, P.O. Box 43640-00100, Nairobi, Kenya; 2 WorldWide Antimalarial Resistance Network, Oxford, UK; 3 Centre for Tropical Medicine and Global Health, University of Oxford, Oxford, UK; 4 Division of National Malaria Programme, Ministry of Health, Nairobi, Kenya

**Keywords:** Malaria, Case-management, Factors, ‘Test and Treat’, Compliance, Kenya

## Abstract

**Background:**

Health workers’ compliance with outpatient malaria ‘test and treat’ guidelines has improved since 2010 but plateaued from 2014 at suboptimal levels in Kenya. This study examined the factors associated with high but suboptimal compliance levels at facilities with available malaria tests and drugs.

**Methods:**

Data from four national, cross-sectional health facility surveys undertaken between 2014 and 2016 in Kenya were analysed. Association between 31 factors and compliance with malaria testing (survey range (SR): 65–69%) and no anti-malarial treatment for test negative patients (SR: 90–92%) were examined using multilevel logistic regression models.

**Results:**

A total of 2,752 febrile patients seen by 594 health workers at 486 health facilities were analysed. Higher odds of malaria testing were associated with lake endemic (aOR = 12.12; 95% CI: 5.3–27.6), highland epidemic (aOR = 5.06; 95% CI: 2.7–9.5) and semi-arid seasonal (aOR = 2.07; 95% CI: 1.2–3.6) compared to low risk areas; faith-based (FBO)/ non-governmental organization (NGO)-owned compared to government-owned facilities (aOR = 5.80; 95% CI: 3.2–10.6); health workers’ perception of malaria endemicity as high-risk (aOR = 3.05; 95% CI: 1.8–5.2); supervision with feedback (aOR = 1.84; 95% CI: 1.2–2.9); access to guidelines (aOR = 1.96; 95% CI: 1.1–3.4); older patients compared to infants, higher temperature measurements and main complaints of fever, diarrhoea, headache, vomiting and chills. Lower odds of testing were associated with febrile patients having main complaints of a cough (aOR = 0.65; 95% CI: 0.5–0.9), a rash (aOR = 0.32; 95% CI: 0.2–0.7) or a running nose (aOR = 0.59; 95% CI: 0.4–0.9). Other factors associated with compliance with test negative results included the type of diagnostic test available at the facility, in-service training, health workers’ age, and correct knowledge of the targeted treatment policy.

**Conclusions:**

To optimize outpatient malaria case-management, reduce testing compliance gaps and eliminate overtreatment of test negative patients, there is a need to focus on compliance within low malaria risk areas in addition to ensuring the universal and continuous availability of ‘test and treat’ commodities. Targeting of older and government health workers; dissemination of updated guidelines; and continuing with in-service training and supportive supervision with feedback is essential. Lastly, there is a need to improve health workers’ knowledge about malaria testing criteria considering their perceptions of endemicity.

**Supplementary Information:**

The online version contains supplementary material available at 10.1186/s12936-022-04093-x.

## Background

Significant improvements in malaria control have been made globally, but progress has plateaued in recent years [1]. Malaria case-management based on parasitological confirmation of all suspected malaria patients before targeted treatment of only test positive cases with artemisinin-based combination therapy (ACT), also known as the ‘test and treat policy, has been a mainstay of malaria control in Africa since 2010 [2]. While the availability of ‘test and treat’ commodities is the prerequisite for the policy implementation [3], health workers’ compliance with recommended guidelines determines the cost-effectiveness of case-management [4, 5] and sets a foundation for effective surveillance [6]. Recent improvements in malaria ‘test and treat’ compliance has been reported across health facilities in Africa [7–10]. Despite the improvement trends, non-compliant practices have not been eliminated, and they are more pronounced with respect to testing than treatment compliance [11–15].

Various factors, either interventional (e.g., training, guidelines, supervision) or non-interventional (e.g., patients’ age, gender, the severity of illness), may influence health workers’ compliance [16–18]. According to Rowe’s framework of determinants, the factors affecting performance can include the health system and facility characteristics, health worker characteristics, patient factors, and characteristics of the broader political and socio-economic environment [17]. Several studies across Africa examined factors associated with health workers’ compliance with malaria ‘test and treat’ guidelines; however, most of these studies were undertaken during the early phases of ACT and diagnostic policy change, characterized by low-performance levels [10, 19–24].

In 2010, Kenya adopted the ‘test and treat’ policy for malaria [25] and implemented a series of programmatic activities to aid the translation of the policy into clinical practice as previously described [26]. Since then, health workers’ compliance with the ‘test and treat’ guidelines has been monitored in the outpatient departments. The primary monitoring indicator has been the composite ‘test and treat’ defined as a febrile patient tested for malaria and artemether-lumefantrine (AL) prescription for test positive or no anti-malarial treatment for test negative patients and major improvements have been reported between 2010 and 2016 [20, 27, 28]. Previous analyses have reported the 2010–2016 compliance trends by malaria endemicity [29] and examined a wide range of factors associated with the major improvement trends over seven years [26]. Malaria endemicity, availability of rapid diagnostic tests (RDTs), and health workers access to guidelines were the main predictors of the improvement trends [26].

Even though one of the strategic objectives is to manage 100% of suspected malaria cases according to the Kenya malaria treatment guidelines by 2023 [30], compliance had plateaued between 2014 and 2016 at high but suboptimal levels. The composite ‘test and treat’ performance plateaued between 60 and 65%. The specific indicators contributing to the plateaued performance were malaria testing ranging between 65 and 69% and no anti-malarial treatment for test negative patients between 90 and 92% [28]. Previous analysis focused on the trends during the improvement period. During the plateau phase, separate analyses are required to define the most important time-matched and current factors influencing the compliance, information necessary to inform revised strategies and interventions to maximize performance. In the current study on the plateau phase, malaria endemicity and various health facility, health worker and patient-level factors were analysed to examine the predictors of compliance at the highest, plateaued performance levels between 2014 and 2016.

## Methods

### Data sources

The Kenyan Division of National Malaria Program (DNMP) has undertaken national, biannual cross-sectional health facility surveys since 2010 to monitor the implementation of the outpatient ‘test and treat’ policy. Detailed descriptions of the sampling, methods and basic descriptions of these data are provided elsewhere [26, 29]. In summary, a proportionate, stratified random sample of health facilities were selected from the national master health facility list taking into consideration the facility type, ownership, and administrative boundaries to ensure national representativeness during each survey round. At each of the surveyed facilities, data collection methods included health facility assessments, interviews with health workers and exit interviews with all eligible outpatients during one survey day when they were ready to leave the facility using structured questionnaires. The patients’ exit interviews included all non-referred and non-pregnant patients weighing > 5 kg across all age groups and presenting for an initial visit with fever or a history of fever. Information was collected from patient-held cards about malaria tests requested, test results reported, treatment prescribed, and direct questioning about patients’ demographics, presenting symptoms, and prior use of anti-malarials. Each facility was assessed to determine the availability of medicines and diagnostics services (RDTs or microscopy). Additionally, febrile patients’ caseload on the survey day, ownership, retrospective stockouts of malaria commodities, and the availability of support tools like malaria treatment guidelines and job aids were also assessed. Finally, all health workers who provided clinical consultations in the outpatient departments were interviewed. Information on their demographic characteristics, outpatient responsibility, pre-service training, access to guidelines and job aids, in-service training, supervision, knowledge about malaria ‘test and treat’ policy and their perceptions of malaria endemicity was collected. Data quality was assured through five days of training of the field workers, double-entry into a Microsoft Access database, and comparisons of data files using a verification program in Microsoft Access and referring to paper-based questionnaires. The present study includes a total of 4 national health facility surveys involving 2,752 febrile patients seen by 594 health workers at 486 health facilities with available ‘test and treat’ commodities undertaken between 2014 and 2016.

### Outcomes and factors

**Fig. 1:**
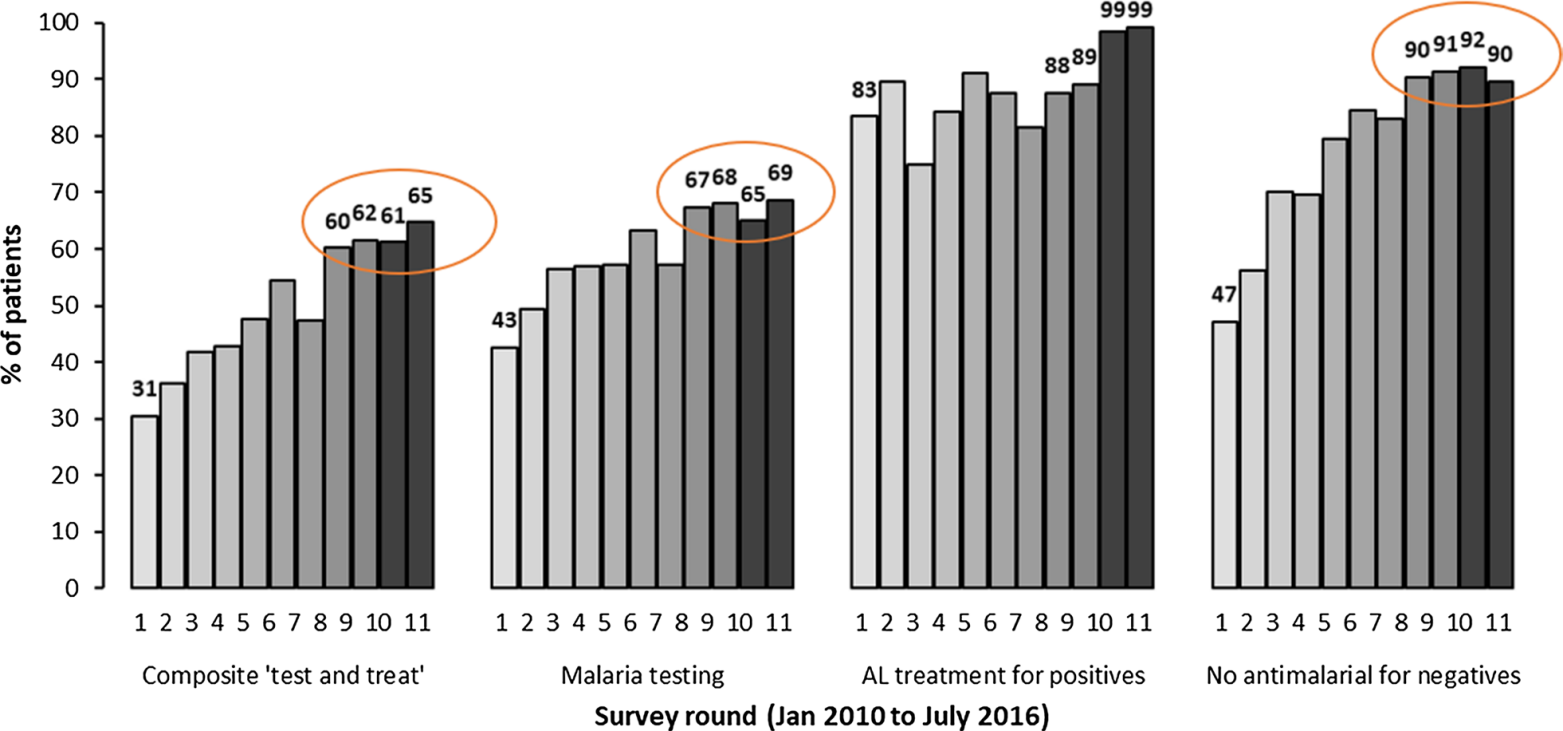
2010–2016 national trends in health workers’ compliance with malaria ‘test and treat’ guidelines. *The trends are from facilities with available malaria diagnostic services and AL in stock showing the plateauing performance levels between 2014 and 2016 circled in orange

During the four surveys undertaken between 2014 and 2016, factors associated with compliance with malaria testing and no anti-malarial treatment for test negative patients were assessed (Fig. 1). The factors associated with AL treatment for test positive patients where health workers’ compliance was nearly optimal (99%) were not assessed. Malaria endemicity, ten health facility, 13 health worker, and six patient-level factors were examined for the association with the 2014–2016 health workers’ compliance. The examined factors varied across the two outcomes based on hypothesized effects and previous literature.

### Statistical analysis

Since the absence of commodities precludes compliance with guidelines, the analysis was restricted to patients who visited facilities with malaria diagnostic services and AL available on the survey day. Due to the lack of significant time trends between 2014 and 2016, a pooled analysis of factors associated with compliance with outcome indicators across the four surveys was undertaken. Multilevel logistic regression models at the patient level with random intercepts at the health facility level to adjust for clustering [31] was performed. The models did not adjust for clustering at the health worker level as the number of health workers interviewed per facility ranged between 1 and 2. For each indicator, the model was specified as follows:

$${\text{logit }}\left( {\mu_{ij} } \right) = \alpha + \beta X_{ij} + \varepsilon_{ij} + \mu_{j}$$

where *μ*_*ij*_ is a probability of compliance with guidelines for that indicator and *X*_*ij*_ is a vector of patient, health worker and health facility factors for patient *i* at health facility *j,* α is the vector of intercepts; *X* is the factor; *β* is the vector of coefficients (factor effects) of the factor on health workers’ compliance at health facility *j* and; *εij* and *µj* the residuals at levels 1 (patient) and 2 (health facility), respectively, and capture unexplained variation.

The final multivariable model was developed for each indicator using the strategy proposed by Collett [32]. First, each covariate was examined in the univariable analysis. Likelihood ratio tests comparing univariable and null models at a significance level of p < 0.05 were used to identify covariates for the initial inclusion in the multivariable model. Any covariates which lost their significance in the presence of other covariates (Wald test, p > 0.05) were then removed from the model. Finally, any other covariates that were non-significant in the univariable analysis or were excluded from the multivariable model were tested one at a time and added to the model in the forward stepwise fashion using the likelihood ratio test at p < 0.05. The process of adding and dropping covariates in the multivariable models was repeated until no more factors could be added to or removed from the models using the likelihood ratio test of p < 0.05. Collinearity between covariates included in the multivariable models was assessed using correlation coefficients in Stata, and collinear variables were omitted when warranted. The association between factors and outcomes were expressed as odds ratios with accompanying 95% CI and p-values in univariable (OR) and multivariable models (aOR). All analyses were conducted using Stata version 15 (StataCorp, College Station, TX, USA).

## Results

### Description of the study population

A total of 2,752 (survey range [SR]: 610–741) febrile patients seen by 594 (SR: 131–156) health workers at 486 (SR: 109–129) health facilities were analysed between 2014 and 2016. Median patients’ age ranged across the surveys between six and eight years. Most of the patients were seen in the lake endemic zone, at government-owned facilities and in dispensaries. Half of the patients were seen by male health workers, 52.4% by nurses and 41.0% by clinical or medical officers (Table 1).

**Table 1:** Key characteristics of interviewed febrile patients in Kenya; 2014–2016 (N = 2752)

		n	%
Malaria endemicity	Epidemiological zone		
	Lake endemic	888	32.3
	Coast endemic	270	9.8
	Highland epidemic	576	20.9
	Semi-arid seasonal	619	22.5
	Low risk	399	14.5
Health facility level	Facility ownership		
	FBO/NGO^a^	299	10.9
	Government	2453	89.1
	Facility level		
	Dispensary	1563	56.8
	Health centre	769	27.9
	Hospital	420	15.3
Health worker level	HW^b^ age		
	≤ 35 years	1754	63.7
	> 35 years	964	35.0
	Missing	34	1.2
	HW gender		
	Male	1376	50.0
	Female	1376	50.0
	Cadre		
	Others^c^	182	6.6
	Nurse	1443	52.4
	Clinical / Medical officer	1127	41.0
	MCM^d^ in-service training		
	No	1057	38.4
	Yes	1695	61.6
	MCM supervision in the previous 3 months		
	No	1571	57.1
	Yes	1181	42.9
	Access to current malaria case-management guidelines		
	No	929	33.8
	Yes	1803	65.5
	Missing	20	0.7
Patient level	Age		
	0–11 months	270	9.8
	12–59 months	845	30.7
	5–14 years	742	27.0
	≥ 15 years	895	32.5
	Gender		
	Male	1223	44.4
	Female	1529	55.6
	Temperature		
	< 37.5 °C	1897	68.9
	≥ 37.5 °C	841	30.6
	Missing	14	0.5

^a^FBO/NGO-Faith-Based/Non-Governmental Organization

^b^HW-Health Worker

^c^Other cadre includes community health workers, nurse aides, laboratory technologists, public health officers, students and support staff

^d^MCM-Malaria Case-Management

### Factors associated with compliance with malaria testing of febrile patients

The proportion of febrile patients tested for malaria between 2014 and 2016 ranged between 66 and 69%. The results from the unadjusted univariable analysis are presented in Additional file 1. From the final multivariable model (Table 2), febrile patients from lake endemic (aOR = 12.12; 95% CI: 5.3–27.6), highland epidemic (aOR = 5.06; 95% CI: 2.7–9.5) and semi-arid seasonal (aOR = 2.07; 95% CI: 1.2–3.6) areas had higher odds of being tested for malaria compared to low risk areas. Similarly, the odds of febrile patients who visited FBO/NGO-owned facilities (234/299,78%) of being tested for malaria were five times more than in government-owned facilities (1607/2453, 66%) (aOR = 5.80; 95% CI: 3.2–10.6).

**Table 2:** Factors associated with compliance with malaria testing of febrile patients, 2014–2016

	Factor	N	Proportion tested n (%)	Unadjusted OR (95% CI)	P-value	Adjusted OR (95% CI)	P-value
Malaria endemicity	Epidemiological zone						
	Low risk	399	141 (35.3)	Ref	< 0.001	Ref	
	Lake endemic	888	797 (89.8)	35.54 (19.0–66.5)	0.002	12.12 (5.3–27.6)	< 0.001
	Coast endemic	270	166 (61.5)	3.26 (1.5–6.9)	< 0.001	2.03 (0.9–4.7)	0.096
	Highland epidemic	576	421 (73.1)	7.08 (3.9–12.8)	0.009	5.06 (2.7–9.5)	< 0.001
	Semi-arid seasonal	619	316 (51.1)	2.06 (1.2–3.5)		2.07 (1.2–3.6)	0.011
Health facility level	Facility ownership						
	Government	2453	1607 (65.5)	Ref		Ref	
	FBO/NGO^a^	299	234 (78.3)	4.99 (2.5–10.1)	< 0.001	5.80 (3.2–10.6)	< 0.001
Health worker level	HW^b^ perception of endemicity						
	Low	1302	630 (48.4)	Ref		Ref	
	High	1446	1209 (83.6)	9.00 (6.0–13.6)	< 0.001	3.07 (1.8–5.3)	< 0.001
	MCM^c^ supervision with feedback in the previous 3 months						
	No	1842	1109 (60.2)	Ref		Ref	
	Yes	910	732 (80.4)	3.20 (2.0–5.1)	< 0.001	1.84 (1.2–2.8)	0.007
	Access to MCM/IMCI^d^ guidelines						
	No	354	231 (65.3)	Ref			
	Yes	2397	1610 (67.2)	1.78 (1.0–3.3)	0.069	1.95 (1.1–3.4)	0.017
Patient-level	Patient age						
	0–11 months	270	133 (49.6)	Ref		Ref	
	12–59 months	845	537 (63.6)	2.22 (1.5–3.3)	< 0.001	2.02 (1.3–3.1)	0.001
	5–14 years	742	558 (75.2)	3.19 (2.1–4.8)	< 0.001	1.56 (1.0–2.5)	0.058
	≥ 15 years	895	613 (68.5)	2.54 (1.7–3.8)	< 0.001	1.54 (1.0–2.5)	0.068
	Temperature						
	< 37.5 °C	1897	1208 (63.7)	Ref		Ref	
	≥ 37.5 °C	841	624 (74.2)	2.09 (1.6–2.7)	< 0.001	2.03 (1.5- 2.7)	< 0.001
	Fever complaint						
	No	353	236 (66.9)	Ref		Ref	
	Yes	2399	1605 (66.9)	1.26 (0.9–1.8)	0.190	2.28 (1.5–3.4)	< 0.003
	Diarrhoea complaint						
	No	2457	1650 (67.2)	Ref		Ref	
	Yes	295	191 (64.8)	1.30 (0.9–1.9)	0.163	1.52 (1.0–2.3)	0.041
	Headache complaint						
	No	1617	947 (58.6)	Ref		Ref	
	Yes	1135	894 (78.8)	2.78 (2.2–3.6)	< 0.001	2.70 (2.0–3.6)	< 0.001
	Vomiting complaint						
	No	2263	1461 (64.6)	Ref		Ref	
	Yes	489	380 (77.7)	2.21 (1.6–3.1)	< 0.001	2.01 (1.4–2.9)	< 0.001
	Chills complaint						
	No	2533	1645 (64.9)	Ref		Ref	
	Yes	219	196 (89.5)	3.51 (2.0–6.2)	< 0.001	3.29 (1.8–6.0)	< 0.001
	Cough complaint						
	No	1521	1107 (72.8)	Ref		Ref	
	Yes	1231	734 (59.6)	0.46 (0.4–0.6)	< 0.001	0.65 (0.5–0.9)	0.001
	Running nose complaint						
	No	2415	1678 (69.5)	Ref		Ref	
	Yes	337	163 (48.4)	0.36 (0.3–0.5)	< 0.001	0.59 (0.4–0.9)	0.005
	Rash complaint						
	No	2687	1813 (67.5)	Ref		Ref	
	Yes	65	28 (43.1)	0.28 (0.1–0.6)	0.001	0.32 (0.2–0.7)	0.003

^a^FBO/NGO- Faith-Based/Non-Governmental Organization

^b^HW-health worker

^c^MCM-malaria case-management

^d^IMCI-Integrated Management of Childhood Illnesses

At the health worker level, febrile patients seen by health workers who perceived malaria endemicity as high risk (aOR = 3.05; 95% CI: 1.8–5.2), who were supervised and received feedback (aOR = 1.84; 95% CI: 1.2–2.9) or had access to either Integrated Management of Childhood Illnesses (IMCI) or malaria case-management guidelines (aOR = 1.96; 95% CI: 1.1–3.4) had higher odds of being tested for malaria compared to those who did not. Febrile patients aged between one to five years compared to infants (aOR = 2.02; 95% CI: 1.3–3.1), those with a temperature measurement of ≥ 37.5 °C (aOR = 2.03; 95% CI: 1.5–2.7), those presenting with a main complaint of fever (aOR = 2.28; 95% CI: 1.5–3.4), diarrhoea (aOR = 1.52; 95% CI: 1.0–2.3), headache (aOR = 2.70; 95% CI: 2.0–3.6), vomiting (aOR = 2.01; 95% CI: 1.4–2.9), or chills (aOR = 3.29; 95% CI: 1.8–6.0) also had higher odds of being tested for malaria. However, febrile patients with a main complaint of a cough (aOR = 0.65; 95% CI: 0.5–0.9), a running nose (aOR = 0.59; 95% CI: 0.4–0.9) or a rash (aOR = 0.32; 95% CI: 0.2–0.7) had significantly lower odds of being tested (Table 2).

### Factors associated with compliance with no anti-malarial treatment for test negative patients

The proportion of test negative patients not treated with an anti-malarial ranged between 90 and 92% between 2014 and 2016. The results from the unadjusted univariable analysis are presented in Additional file 2. There were no differences in compliance across malaria endemicity zones. From the final multivariable model (Table 3) only one facility-level factor, the type of diagnostic test, was associated with compliance. Patients who visited facilities with microscopy only compared to RDTs only available on the survey day as the malaria diagnostic method had 68% lower odds of not being given anti-malarials when they tested negative (aOR = 0.32; 95% CI: 0.1–0.9).

**Table 3:** Factors associated with compliance with no anti-malarial treatment for test negative patients, 2014–2016

	Factor	N	Proportion not treated n (%)	Unadjusted OR (95% CI)	P-value	Adjusted OR (95% CI)	P-value
Health facility level	Type of malaria diagnostic at the facility						
	RDTs^a^	479	443 (92.5)	Ref		Ref	
	Microscopy	212	180 (84.9)	0.36 (0.1- 1.0)	0.054	0.32 (0.1–0.9)	0.031
	Both	390	369 (94.6)	1.83 (0.7–5.1)	0.249	2.27 (0.8–6.4)	0.120
Health worker level	HW^b^ age						
	≤ 35 years	680	628 (92.4)	Ref		Ref	
	> 35 years	387	350 (90.4)	0.47 (0.2–1.1)	0.074	0.28 (0.1–0.7)	0.005
	HW perception of endemicity				0.003		< 0.001
	Low	540	512 (94.8)	Ref		Ref	
	High	539	478 (88.7)	0.27 (0.1–0.7)		0.16 (0.1–0.4)	
	Correct knowledge on malaria treatment policy						< 0.001
	No	116	85 (73.3)	Ref	< 0.001	Ref	
	Yes	965	907 (94.0)	10.32 (4.0–26.6)	< 0.001	8.89 (3.2–25.1)	< 0.001
	MCM^c^ in-service training						
	No	422	365 (86.5)	Ref		Ref	
	Yes	659	627 (95.1)	5.49 (2.5–12.2)	0.105	5.34 (2.2–12.9)	0.013
Patient-level	Temperature			0.79 (0.6–1.1)	0.002	0.69 (0.5–0.9)	0.035
	Headache main complaint						
	No	623	584 (93.7)	Ref		Ref	
	Yes	458	408 (89.1)	0.38 (0.2–0.7)		0.51 (0.3–1.0)	0.002
	Cough main complaint						
	No	574	510 (88.9)	Ref		Ref	
	Yes	501	482 (95.1)	3.31 (1.7–6.6)	0.001	2.91 (1.5–5.8)	

^a^RDTs-Rapid diagnostic tests

^b^HW-health worker

^c^MCM-malaria case-management

At the health worker level, lower odds of compliance were observed among patients seen by older health workers (aOR = 0.28; 95% CI: 0.1–0.7) and those who perceived the endemicity as high-risk (aOR = 0.16; 95% CI: 0.1–0.4). Patients seen by health workers who were knowledgeable about the targeted malaria treatment policy (aOR = 8.89; 95% CI: 3.2–25.1) and by those who had received in-service training (aOR = 5.34; 95% CI: 2.2–12.9) had higher odds of correct management compared to those who did not. A unit increase in temperature measurement (aOR = 0.69; 95% CI: 0.5–0.9) and the main complaint of headache (aOR = 0.51; 95% CI: 0.3–0.9) were associated with lower odds, while the main complaint of a cough led to 2.9 times higher odds of compliance (aOR = 2.91; 95% CI: 1.5–5.8) (Table 3).

## Discussion

Drawing on Rowe’s framework of the determinants of health worker performance [17], this study revealed various interventional and non-interventional factors affecting ‘test and treat’ compliance at high but suboptimal levels between 2014 and 2016 in Kenya. The factors were often different from those associated with the compliance trends moving from low to high levels of performance from a previous study within the same areas [26]. A total of 23 factors were identified across the two periods (the improvement and plateau performance periods), with only eight associated with compliance across both periods, while 12 more factors were identified in this analysis.

Understanding the effect of interventional factors on compliance is imperative to discerning what works to address non-interventional factors and the case-management gaps. In addition to RDT availability and health workers’ access to malaria case-management and IMCI guidelines also identified in the previous analysis [26], training and supervision with feedback were associated with compliance during this period. Health workers were less likely to comply with test negative results at facilities where malaria diagnosis was exclusively based on microscopy than those providing malaria testing by only RDTs. Lack of confidence in microscopy results due to quality issues [33, 34] and gained confidence over the years in RDT results [35, 36] might explain the observed association. Even though the positive association between availability of RDTs with compliance with test negative results supports the Kenyan policy decision on introducing RDTs exclusively at health facilities without diagnostic capacities [37], the lack of association observed on malaria testing during this plateauing performance phase calls for further research to understand health workers’ testing behaviour.

Furthermore, the most widely used interventions to improve health worker performance (i.e., training and supervision with feedback) were associated with compliance during this period. In-service training on the new malaria case-management policy was associated with up to five times higher odds of treatment compliance with test negative patients, while supervision with feedback was associated with higher malaria testing. The finding contrasts with the lack of association of these two interventions with compliance reported in Kenya at low levels of performance [20], during the 2010–2016 improvement trends [26] and from cross-sectional studies across other African countries [10, 19, 21]. Although qualitative research is required, it can be hypothesized that the quality of supportive supervision and in-service training improved over time and resulted in significant associations observed only at the highest levels of performance.

Of importance to note is the lack of association between in-service training and malaria testing, where a major gap in compliance remains. This might imply that training might cease to be an effective intervention once compliance with test negative results is optimized. Moreover, the lack of association between these two interventions and compliance at low-performance levels and during the improvement trends raises the need to understand their quality, content, and delivery qualitatively. Nevertheless, the positive associations found at these high levels call for the use of multifaceted interventions incorporating training and supervision with feedback to optimize health worker performance [38–42].

Another common intervention, dissemination of malaria case-management guidelines, was associated with higher malaria testing. A finding similar to a report from Malawi where health workers who had access to guidelines were more likely to test for malaria at higher compliance levels of 76% [9] and in contrast to a Kenyan study that did not find an association when testing rates were low [20]. This finding suggests continued and strengthened dissemination of the guidelines to health workers.

Non-interventional factors, particularly patient characteristics, were strong predictors of compliance during this period. Although these factors are not directly modifiable, NMCPs, and other implementing partners should consider them when implementing the interventions to improve compliance. Health workers seem to rely on signs and symptoms to rule out malaria in febrile patients despite the poor sensitivity of various clinical case definitions [43]. Patients’ main complaints traditionally related to uncomplicated malaria (e.g., fever, headache, vomiting, chills, and diarrhoea) were associated with higher malaria testing, whereas complaints less suggestive of malaria (e.g., cough, rash, and running nose) resulted in lower testing compliance. In contrast, higher body temperature negatively influenced compliance with test negative results, while a cough increased the odds of compliance with test negative results by up to three times. The findings are similar to other studies that indicated patients’ clinical signs and symptoms influence compliance with malaria guidelines [9, 19, 20, 23, 24, 44]. Given the strong association between compliance and various signs and symptoms, the strategy of encouraging patients to report fever or a history of fever spontaneously to health workers should be explored. On the other hand, health workers should be reminded to ask patients about fever, measure temperature, test all febrile patients regardless of other presenting symptoms or be informed of the possibility of coinfections during training (e.g., malaria on top of an acute respiratory illness).

Malaria endemicity continued to independently influence health workers’ compliance with the outpatient malaria case-management guidelines as previously reported while analysing the 2010–2016 trends [26, 29]. Notably, higher malaria risk was associated with malaria testing of febrile patients as similarly reported in Angola [10]. The findings further highlight the need to target interventions aimed at improving case-management practices in low malaria risk areas. Moreover, health workers’ perception of malaria endemicity was independently associated with compliance during this period. As also speculated in Mozambique [22], the perception of an area as high risk was also a strong predictor increasing the likelihood of testing by three times. The probabilistic reasoning applied before testing febrile patients for malaria may explain the observed pattern [45, 46]. Similarly, health workers’ perception of malaria endemicity as high was associated with overtreatment of test negative patients for malaria. While no study has examined the association between overtreatment and perception of risk, the common explanations for this behaviour might include mistrust of test results [11, 47], a long history of presumptive treatment, or lack of alternative diagnosis [48]. Health workers’ perception of malaria endemicity should be considered when implementing interventions to improve compliance.

Another factor that continued to influence compliance was the ownership of health facilities, with FBO/NGO-owned facilities having a higher likelihood of malaria testing than government facilities. The probable reasons for higher testing policy adoption in the FBO/NGO sector might include the higher cost of laboratory services [49], wealthier patients [20], and higher motivation of health workers [50] in this sector. The findings call for further targeting of government health workers with effective strategies to improve their compliance. Additionally, correct health workers’ knowledge about the targeted treatment policy continued to be a predictor of compliance with negative test results, as previously reported [26]. The findings support continued in-service training of health workers to expand their knowledge. Lastly, at these high-performance levels, health workers’ age was associated with compliance, with older health workers less likely to comply with no anti-malarial treatment policy for test negative patients. The reliance on their experiences more than guidelines or resistance to change among older health workers could explain the observed pattern, as reported in an evaluation of the quality of IMCI care in Benin [51].

This study has some limitations. As health workers’ behaviour might be affected by various factors, including contextual and latent ones (e.g., motivation, attitude, experience) that could not be examined from the data, further qualitative research is needed to close the testing and negative test compliance gaps. As with all large, multivariable datasets with multiple comparisons of factors, some of the results may have been significant by chance. The data series included in this analysis ended in 2016, factors influencing health worker adherence to guidelines may have changed since, it is important to sustain surveys of performance longitudinally to continuously inform case-management practices.

## Conclusion

Malaria case-management should be longitudinally monitored as health workers’ compliance might reach a plateau without further improvements. During such a plateau, analyses of the current factors influencing compliance are required to inform the tailoring of interventions to improve compliance further. The package of interventions that the Kenyan DNMP can consider to optimize compliance might include constant supply and availability of RDTs, dissemination of malaria case-management and IMCI guidelines, high-quality in-service training and supervision with feedback. The implementation of the interventions should focus on the compliance gaps and health worker performance in low malaria risk areas; target older and government health workers; and generally, improve health workers’ knowledge about malaria testing criteria considering their perceptions of endemicity.

As the greatest gap remains in compliance with malaria testing, especially within low malaria risk areas where malaria has been targeted for elimination in some counties, there is a need for further qualitative research to provide an in-depth understanding of the determinants of compliance with malaria testing, describe the quality and content of training and supervision routinely delivered to health workers, and develop and test interventional studies identifying the most cost-effective set of strategies for further improvement.

## Supplementary Information

**Additional file 1**. Univariable analysis of factors associated with compliance with malaria testing of febrile patients, 2014–2016.

**Additional file 2**. Univariable analysis of factors associated with compliance with no antimalarial for test negative patients, 2014–2016.

## Data Availability

The dataset supporting the conclusions of this article cannot be shared publicly because they are under the ownership of the Kenyan Division of National Malaria Program (DNMP) and the KEMRI-Wellcome Trust Research Programme. Therefore, the data used are available upon request by submitting a letter indicating the proposed use and justification to the Director of the Kenyan DNMP and through a formal requesting process to the KEMRI Institutional Data Access/Ethics Committee. The details of the guidelines can be found on the KEMRI Wellcome website (https://dataverse.harvard.edu/dataverse/kwtrp). Access to data is provided via the KEMRI Wellcome Data Governance Committee: dgc@kemri-wellcome.org.
