# Supplementary material for: Factors influencing health workers’ compliance with outpatient malaria ‘test and treat’ guidelines during the plateauing performance phase in Kenya, 2014–2016
Source: Malar J. 2022 Mar 3;21:68. doi: 10.1186/s12936-022-04093-x (PMC8895910; doi:10.1186/s12936-022-04093-x)
Supplement: Supplementary file 1 — Additional file 1. Univariable analysis of factors associated with compliance with malaria testing of febrile patients, 2014–2016. [file 12936_2022_4093_MOESM1_ESM.docx]

**Additional file 1: Univariable analysis of factors associated with compliance with malaria testing of febrile patients, 2014-2016**

|  | | **Factor** | **N=2752**  **n (%)** | **Tested [n (%)]** | **Unadjusted OR (95% CI)** | **P-value** |
| --- | --- | --- | --- | --- | --- | --- |
| **Malaria endemicity** | | **Epidemiological zone**  Low risk  Lake endemic  Coast endemic  Highland epidemic  Semi-arid seasonal | 399 (14.5)  888 (32.3)  270 (9.8)  576 (20.9)  619 (22.5) | 141 (35.3)  797 (89.8)  166 (61.5)  421 (73.1)  316 (51.1) | Ref  35.54 (19.0 - 66.5)  3.26 (1.5 - 6.9)  7.08 (3.9 - 12.7)  2.06 (1.2 – 3.5) | **<0.001**  **0.002**  **<0.001**  **0.009** |
| **Health facility level** | | **Facility ownership**  Government  FBO/NGO^a^ | 2453 (89.1)  299 (10.9) | 1607 (65.5)  234 (78.3) | Ref  4.99 (2.5 – 10.1) | **<0.001** |
|  | | **Facility level**  Dispensary  Health centre  Hospital | 1563 (56.8)  769 (27.9)  420 (15.3) | 1079 (69.0)  512 (66.6)  250 (59.5) | Ref  1.01 (0.6 - 1.7)  0.78 (0.5 - 1.3) | 0.965  0.164 |
|  | | **Caseload on the survey day**  ≤25 patients  >25 patients | 2582 (93.8)  170 (6.2) | 1694 (65.6)  147 (86.5) | Ref  3.57 (0.6 - 20.4) | 0.153 |
|  | | **Type of malaria diagnostic at the facility**  RDTs^b^  Microscopy  Both RDT & microscopy | 1315 (47.8)  573 (20.8)  864 (31.4) | 858 (62.3)  375 (65.5)  608 (70.4) | Ref  1.84 (1.0 - 3.5)  1.98 (1.0 - 3.7) | 0.061  **<0.013** |
|  | | **Retrospective RDT stockouts**  No  Yes | 1978 (71.9)  739 (26.9) | 1309 (66.2)  513 (69.4) | Ref  1.93 (1.1 - 3.3) | **0.020** |
|  | | **Retrospective microscopy stockouts**  No  Yes | 2068 (75.2)  684 (24.9) | 1391 (67.3)  450 (65.8) | Ref  0.72 (0.4 - 1.2) | 0.226 |
|  | | **Retrospective RDT and microscopy stockouts**  No  Yes | 2644 (96.1)  108 (3.9) | 1749 (66.2)  92 (85.2) | Ref  6.85 (1.7 - 27.3) | **0.006** |
|  | | **Retrospective AL^c^ stockouts**  No  Yes | 1161 (42.2)  1567 (56.9) | 809 (69.7)  1020 (65.1) | Ref  0.60 (0.4 – 1.0) | **0.041** |
|  | | **Malaria guidelines available**  No  Yes | 1883 (69.0)  847 (31.0) | 574 (67.8)  1252 (66.5) | Ref  1.09 (0.7 - 1.8) | 0.733 |
|  | | **Malaria new chart**  No  Yes | 2001 (72.7)  719 (26.1) | 1345 (67.2)  474 (65.9) | 1.08 (0.7 - 1.8)  Ref | 0.763 |
| **Health worker level** | **HW age**  ≤35 years  >35 years | | 1754 (64.5)  964 (35.5) | 1159 (66.1)  650 (67.4) | Ref  0.90 (0.6 - 1.4) | 0.617 |
|  | **HW^e^ gender**  Female  Male | | 1376 (50.0)  1376 (50.0) | 911 (66.2)  930 (67.6) | Ref  1.02 (0.7- 1.5) | 0.934 |
|  | **Facility in charge**  No  Yes | | 1779 (65.3)  947 (34.7) | 1155 (64.9)  670 (70.8) | Ref  1.24 (0.8 - 1.9) | 0.761 |
|  | **Cadre**  Others  Nurse  Clinical officer/ Medical officer | | 182 (6.6)  1443 (52.4)  1127 (41.0) | 125 (68.7)  988 (68.5)  728 (64.6) | Ref  1.03 (0.4 - 2.4)  1.10 (0.5 - 2.6) | 0.938  0.835 |
|  | **HW perception of malaria risk**  Low  High | | 1302 (47.4)  1446 (52.6) | 630 (48.4)  1209 (83.6) | Ref  9.00 (6.0 - 13.6) | **<0.001** |
|  | **MCM^f^ in-service training**  No  Yes | | 1057 (38.4)  1695 (61.6) | 674 (63.8)  1167 (68.9) | Ref  1.18 (0.8 - 1.7) | 0.379 |
|  | **Access to current malaria diagnosis and treatment guidelines**  No  Yes | | 929 (34.0)  1803 (66.0) | 645 (69.4)  1186 (65.8) | Ref  1.19 (0.7 - 1.9) | 0.481 |
|  | **Access to IMCI^g^ guidelines**  No  Yes | | 818 (29.8)  1929 (70.2) | 544 (66.5)  1296 (67.2) | Ref  1.05 (0.7-1.6) | 0.816 |
|  | **Any supervision in the previous 3 months**  No  Yes | | 866 (31.5)  1886 (68.5) | 512 (59.1)  1329 (70.5) | Ref  1.59 (1.0 - 2.5) | **0.036** |
|  | **MCM supervision in the previous 3 months**  No  Yes | | 1571 (57.1)  1181 (42.9) | 937 (59.6)  904 (76.6) | Ref  2.61 (1.7 – 4.0) | **<0.001** |
|  | **Observation of consultations in the previous 3 months**  No  Yes | | 2044 (74.3)  708 (25.7) | 1287 (63.0)  554 (78.3) | Ref  2.90 (1.7 - 4.9) | **<0.001** |
|  | **Feedback in the previous 3 months**  No  Yes | | 1842 (66.9)  910 (33.1) | 1109 (60.2)  732 (80.4) | Ref  3.20 (2.0 - 5.1) | **<0.001** |
|  | **Correct knowledge on testing policy**  No  Yes | | 268 (9.7)  2484 (90.3) | 135 (50.4)  1706 (68.7) | Ref  1.84 (1.1 - 3.2) | **0.030** |
| **Patient level** | **Patient age**  0-11 months  12-59 months  5-14 years  ≥15 years | | 270 (9.8)  845 (30.7)  742 (27.0)  895 (32.5) | 133 (49.3)  537 (63.6)  558 (75.2)  613 (68.5) | Ref  2.22 (1.5 - 3.3)  3.19 (2.1 - 4.8)  2.54 (1.7 - 3.8) | **<0.001**  **<0.001**  **<0.001** |
|  | **Duration of illness (median IQR)** | |  |  | 0.96 (0.9 - 1.0) | 0.093 |
|  | **Temperature**  <37.5°C  ≥37.5°C | | 1897 (69.3)  841 (30.7) | 1208 (63.7)  624 (74.2) | Ref  2.09 (1.6 - 2.7) | **<0.001** |
|  | **Prior use of antimalarial**  No  Yes | | 2663 (96.8)  89 (3.2) | 1768 (66.4)  73 (82.0) | Ref  0.95 (0.8 - 1.1) | 0.540 |
|  | **Main complaints** | |  |  |  |  |
|  | **Fever**  No  Yes | | 353 (12.8)  2399 (87.2) | 236 (66.9)  1605 (66.9) | Ref  1.26 (0.9-1.8) | 0.190 |
|  | **Cough**  No  Yes | | 1521 (55.3)  1231 (44.7) | 1107 (72.8)  734 (59.6) | Ref  0.46 (0.4 - 0.6) | **<0.001** |
|  | **Diarrhoea**  No  Yes | | 2457 (89.3)  295 (10.7) | 1650 (67.2)  191 (64.8) | Ref  1.30 (0.9-1.9) | 0.163 |
|  | **Headache**  No  Yes | | 1617 (58.7)  1135 (41.2) | 947 (58.6)  894 (78.8) | Ref  2.78 (2.2 - 3.6) | **<0.001** |
|  | **Running nose**  No  Yes | | 2415 (87.8)  337 (12.3) | 1678 (69.5)  163 (48.4) | Ref  0.36 (0.3 - 0.5) | **<0.001** |
|  | **Rash**  No  Yes | | 2687 (97.6)  65 (2.4) | 1813 (67.5)  28 (43.1) | Ref  0.28 (0.1 - 0.6) | **0.001** |
|  | **Vomiting**  No  Yes | | 2263 (82.2)  489 (17.8) | 1461 (64.6)  380 (77.7) | Ref  2.21 (1.6 - 3.1) | **<0.001** |
|  | **Chills**  No  Yes | | 2533 (92.0)  219 (8.0) | 1645 (64.9)  196 (89.5) | Ref  3.51 (2.0 - 6.2) | **<0.001** |
|  | **Case complexity**  No fever  Fever only  Fever and other complaints | | 353 (12.8)  315 (11.5)  2084 (75.7) | 236 (66.9)  201 (63.8)  1404 (67.4) | Ref  1.12 (0.7 - 1.8)  1.28 (0.8 - 2.0) | 0.624  0.164 |

^a^ FBO/NGO-Faith-Based/Non-Governmental Organisation; ^b^RDTs-Rapid Diagnostic Tests; ^c^AL-Artemether-Lumefantrine; ^d^IQR-Interquartile range; ^e^HW-Health Worker; ^f^MCM-Malaria Case-Management; ^g^IMCI-Integrated Management of Childhood Illness
