# Supplementary material for: Factors influencing health workers’ compliance with outpatient malaria ‘test and treat’ guidelines during the plateauing performance phase in Kenya, 2014–2016
Source: Malar J. 2022 Mar 3;21:68. doi: 10.1186/s12936-022-04093-x (PMC8895910; doi:10.1186/s12936-022-04093-x)
Supplement: Supplementary file 2 — Additional file 2. Univariable analysis of factors associated with compliance with no antimalarial for test negative patients, 2014–2016. [file 12936_2022_4093_MOESM2_ESM.docx]

**Additional file 2: Univariable analysis of factors associated with compliance with no antimalarial for test negative patients, 2014-2016**

|  | **Factor** | **N=1081**  **n (%)** | **No antimalarial [n (%)]** | **Unadjusted OR (95% CI)** | **P-value** |
| --- | --- | --- | --- | --- | --- |
| **Malaria endemicity** | **Epidemiological zone**  Low risk  Lake endemic  Coast endemic  Highland epidemic  Semi-arid seasonal | 116 (10.7)  306 (28.3)  110 (10.2)  289 (26.7)  260 (24.1) | 114 (98.3)  274 (89.5)  109 (99.1)  256 (88.6)  239 (91.9) | Ref  0.07 (0.0 - 0.6)  2.13 (0.1 - 58.7)  0.05 (0.0 - 0.5)  0.09 (0.0 - 0.8) | **0.017**  0.656  **0.008**  **0.026** |
| **Health facility level** | **Facility ownership**  FBO/NGO^a^  Government | 161 (14.9)  920 (85.1) | 147 (91.3)  845 (91.9) | Ref  0.78 (0.3 - 2.7) | 0.696 |
|  | **Facility level**  Dispensary  Health centre  Hospital | 621 (57.5)  302 (27.9)  158 (14.6) | 572 (92.1)  282 (93.4)  138 (87.3) | Ref  1.52 (0.5 - 4.4)  0.52 (0.2 - 1.7) | 0.441  0.273 |
|  | **Type of malaria diagnostic at facility**  RDTs^b^  Microscopy  Both RDT & microscopy | 479 (44.3)  212 (19.6)  390 (36.1) | 443 (92.5)  180 (84.9)  369 (94.6) | Ref  0.36 (0.1 - 1.0)  1.83 (0.7 - 5.1) | **0.054**  0.249 |
|  | **Retrospective RDT stockouts**  No  Yes | 776 (72.6)  293 (27.4) | 721 (92.9)  259 (88.4) | Ref  0.45 (0.2 - 1.1) | 0.090 |
|  | **Retrospective microscopy stockouts**  No  Yes | 799 (73.9)  282 (26.1) | 733 (91.7)  259 (91.8) | Ref  1.22 (0.4 - 3.4) | 0.699 |
|  | **Retrospective RDT and microscopy stockouts**  No  Yes | 1037 (95.9)  44 (4.1) | 952 (91.8)  40 (90.9) | Ref  0.52 (0.1 - 3.7) | 0.513 |
|  | **Retrospective AL^c^ stockouts**  No  Yes | 495 (46.1)  578 (53.9) | 460 (93.9)  524 (90.7) | Ref  0.66 (0.3 - 1.6) | 0.354 |
|  | **Malaria guidelines available**  No  Yes | 316 (29.6)  752 (70.4) | 287 (90.8)  694 (92.3) | Ref  1.31 (0.5 - 3.5) | 0.584 |
|  | **Malaria new chart**  No  Yes | 776 (72.9)  288 (27.1) | 709 (91.4)  266 (92.4) | Ref  1.30 (0.5 - 3.4) | 0.596 |
| **Health worker level** | **HW age**  ≤35 years  >35 years | 680 (63.7)  387 (36.3) | 628 (92.4)  350 (90.4) | Ref  0.47 (0.2 - 1.1) | 0.074 |
|  | **HW gender**  Female  Male | 571 (52.8)  510 (47.2) | 534 (93.5)  458 (89.8) | Ref  0.44 (0.2 – 1.0) | **0.044** |
|  | **Facility in charge**  No  Yes | 696 (65.1)  373 (34.9) | 640 (92.0)  340 (91.2) | Ref  0.79 (0.4 - 1.7) | 0.557 |
|  | **Cadre**  Others  Nurse  Clinical officer/ Medical officer | 77 (7.1)  583 (53.9)  421 (39.0) | 66 (85.7)  542 (93.0)  384 (91.2) | Ref  3.39 (0.9 - 12.9)  2.78 (0.7 - 11.5) | 0.073  0.158 |
|  | **HW^e^ perception of endemicity**  Low  High | 540 (50.1)  539 (50.0) | 512 (94.8)  478 (88.7) | Ref  0.27 (0.1 - 0.7) | **0.003** |
|  | **MCM^f^ in-service training**  No  Yes | 422 (39.0)  659 (61.0) | 365 (86.5)  627 (95.1) | Ref  5.49 (2.5 - 12.2) | **<0.001** |
|  | **Access to current malaria diagnosis and treatment guidelines**  No  Yes | 357 (33.3)  714 (66.7) | 322 (90.2)  660 (92.4) | Ref  1.01 (0.4 - 2.5) | 0.985 |
|  | **Access to IMCI^g^ guidelines**  No  Yes | 312 (28.9)  768 (71.1) | 281 (90.1)  710 (92.5) | Ref  1.46 (0.6-3.4) | 0.381 |
|  | **Any supervision in the previous 3 months**  No  Yes | 353 (32.7)  728 (67.4) | 331 (93.8)  661 (90.8) | Ref  0.52 (0.2 - 1.3) | 0.175 |
|  | **MCM supervision in the previous 3 months**  No  Yes | 640 (59.2)  441 (40.8) | 591 (92.3)  401 (90.9) | Ref  0.87 (0.4 - 2.0) | 0.752 |
|  | **Observation of consultations in the previous 3 months**  No  Yes | 834 (77.2)  247 (22.9) | 766 (91.9)  226 (91.5) | Ref  1.12 (0.4 - 3.1) | 0.829 |
|  | **Feedback in the previous 3 months**  No  Yes | 741 (68.6)  340 (31.5) | 682 (92.0)  310 (91.2) | Ref  1.01 (0.4 - 2.6) | 0.989 |
|  | **Correct knowledge on malaria treatment policy**  No  Yes | 116 (10.7)  965 (89.3) | 85 (73.3)  907 (94.0) | Ref  10.27 (4.0 - 26.4) | **<0.001** |
| **Patient level** | **Patient age**  0-11 months  12-59 months  5-14 years  ≥15 years | 93 (8.6)  338 (31.3)  240 (22.2)  410 (37.9) | 85 (91.4)  314 (92.9)  216 (90.0)  377 (92.0) | Ref  1.10 (0.3 - 3.6)  0.63 (0.2 - 2.2)  0.55 (0.2 - 1.8) | 0.880  0.461  0.326 |
|  | **Duration of illness (median IQR)** | 3 (2-4) |  | 1.00 (0.9 - 1.1) | 0.954 |
|  | **Temperature**  <37.5°C  ≥37.5°C | 769 (71.5)  307 (28.5) | 710 (92.3)  277 (90.2) | Ref  0.69 (0.4 - 1.3) | 0.274 |
|  | **Prior use of antimalarial**  No  Yes | 1038 (96.0)  43 (4.0) | 955 (92.0)  37 (86.1) | Ref  0.37 (0.1 - 1.4) | 0.128 |
|  | **Main complaints** |  |  |  |  |
|  | **Fever**  No  Yes | 134 (12.4)  947 (87.6) | 123 (91.8)  869 (91.8) | Ref  1.20 (0.5 - 3.0) | 0.695 |
|  | **Cough**  No  Yes | 574 (53.1)  507 (46.9) | 510 (88.9)  482 (95.1) | Ref  3.31 (1.7 - 6.6) | **0.001** |
|  | **Diarrhoea**  No  Yes | 945 (87.4)  136 (12.6) | 867 (91.8)  125 (91.9) | Ref  1.39 (0.6 - 3.5) | 0.475 |
|  | **Headache**  No  Yes | 623 (57.6)  458 (42.4) | 584 (93.7)  408 (89.1) | Ref  0.38 (0.2 - 0.7) | **0.002** |
|  | **Running nose**  No  Yes | 961 (88.9)  120 (11.1) | 877 (91.3)  115 (95.8) | Ref  2.54 (0.7 - 8.6) | 0.132 |
|  | **Rash**  No  Yes | 1060 (98.1)  21 (1.9) | 972 (91.7)  20 (95.2) | Ref  0.89 (0.1 - 10.1) | 0.928 |
|  | **Vomiting**  No  Yes | 921 (85.2)  160 (14.8) | 845 (91.8)  147 (91.9) | Ref  1.04 (0.5 - 2.4) | 0.933 |
|  | **Chills**  No  Yes | 991 (91.7)  90 (8.3) | 912 (92.0)  80 (88.9) | Ref  0.51 (0.2 - 1.4) | 0.176 |
|  | **Case complexity**  No fever  Fever only  Fever and other complaints | 134 (12.4)  124 (11.5)  823 (76.1) | 123 (91.1)  116 (93.6)  753 (91.5) | Ref  2.14 (0.6 - 7.8)  1.11 (0.4 - 2.8) | 0.255  0.823 |

^a^ FBO/NGO-Faith-Based/Non-Governmental Organisation; ^b^RDTs-Rapid Diagnostic Tests; ^c^AL-Artemether-Lumefantrine; ^d^IQR-Interquartile range; ^e^HW-Health Worker; ^f^MCM-Malaria Case-Management; ^g^IMCI-Integrated Management of Childhood Illness
